# Safety of an extended-release injectable moxidectin suspension formulation (ProHeart^®^ 12) in dogs

**DOI:** 10.1186/s13071-019-3690-6

**Published:** 2019-09-06

**Authors:** Matthew J. Krautmann, Sean Mahabir, Ann Fielder, Wendy Collard, Tracie L. Wolthuis, Kevin Esch, Tracy Morton, Kent Alleman, Laibin Luo, Erin McCandless, Steven Nederveld, Kristina Kryda, Ryan Carroll, Joseph F. Boucher

**Affiliations:** Zoetis, Kalamazoo, MI 49007 USA

**Keywords:** ProHeart^®^ 12, ProHeart^®^ SR-12, Moxidectin, Safety, Toxicology, Dog

## Abstract

**Background:**

The safety of ProHeart® 12 (PH 12; extended-release injectable suspension; 10% moxidectin in glyceryl tristearate microspheres) was evaluated in four studies using Beagle dogs and one study using ivermectin-sensitive Collies. The recommended dose is 0.5 mg/kg subcutaneously once yearly.

**Methods:**

Study 1: safety margin was evaluated as 3 treatments of PH 12 (0× (control); 1× (recommended dose); 3× (3 times recommended dose) and 5× (5 times recommended dose) in 12 months *via* clinical observations, body weights, food consumption, injection site observations, physical examinations, moxidectin tissue assay, pharmacokinetics, and clinical and anatomic pathology. Study 2: safety in breeding-age males was demonstrated by semen testing at 14-day intervals from Day 7 to Day 91 post-treatment (0× or 3×). Study 3: reproductive safety in females was demonstrated by monitoring dams and litters following treatments (0× or 3×) administered during breeding, gestation, or lactation. Study 4: safety in dogs surgically implanted with adult heartworms was evaluated by clinical and laboratory monitoring following treatment with 0× or 3× administered 61 days post-implantation. Study 5: safety in ivermectin-sensitive dogs (120 µg/kg SC) was by clinical monitoring for 1 week after administering 1×, 3× or 5×.

**Results:**

Study 1: slight swelling clinically detectable at some 3× and 5× injection sites was characterized microscopically as granulomatous inflammation, like tissue responses to medical implants, interpreted as non-adverse. Pharmacokinetics were dose-proportional and there was little or no systemic accumulation. Residual moxidectin mean (range) at 1× injection sites after 1 year was 16.0% (0.045–37.6%) of the administered mass. Studies 2 and 3: no effects were identified in reproductive indices (females) or semen quality characteristics (males). Study 4: PH 12 produced marked reductions in circulating microfilariae and lower numbers of adult heartworms, but no adverse clinical signs were identified. Study 5: there were no abnormal clinical signs at 1×, 3× or 5× overdoses of PH 12 in ivermectin-sensitive dogs.

**Conclusions:**

PH 12 has a > 5× safety margin in both normal and ivermectin-sensitive dogs, has no effects on canine reproduction, and is well tolerated in heartworm-positive dogs. The only treatment-related finding was non-adverse, granulomatous inflammation at the injection site.

## Background

The heartworm preventative ProHeart^®^ SR-12 (PH 12; Zoetis, Parsippany, NJ) is an extended-release microsphere suspension formulation of moxidectin that provides continuous heartworm prophylaxis for 1 year following subcutaneous injection. PH 12 was first registered in Australia in late 2000 and became the top-selling heartworm preventative, with 47% market share by January 2005, due to its reliable effectiveness, superior compliance, and overall clinical safety profile. Over the past 11 years an estimated 12.4 million doses have been distributed there. More recently, PH 12 is also being marketed in a number of Asian and Latin American countries. In the USA, ProHeart^®^ 6 (PH 6) employs the same microsphere suspension as PH 12 at a lower dosage that protects against heartworm disease for 6 months. Shortly after introduction of PH 6 to the USA market, concerns were raised about severe anaphylactoid responses in the first 48 hours post-treatment. In response, the former manufacturer (Fort Dodge Animal Health; Overland Park, KS, USA) initiated an extensive pharmacovigilance (PV) monitoring program, which included agreement to report all adverse reactions. Over the past 10+ years, the close monitoring has demonstrated a stable and predictable PV profile for PH 6 that is very similar to the PV profile of PH 12 internationally and is in line with profiles of other marketed veterinary products. Given the comparability between the PH 6 PV profile and that of PH 12 internationally, that owner non-compliance continues to be an issue with monthly-use heartworm preventative products, and that the once-yearly dose interval for PH12 provides inherently superior compliance, Zoetis elected to seek approval for marketing of PH 12 in the USA. The safety objectives for PH 12 studies outlined in this article and in McTier et al. [1] were extended as feasible to elucidating the origin and incidence of hypersensitivity-related responses if any should be observed.

All ProHeart® (PH) injectable products consist of 10% moxidectin microspheres (90–180 µm) in glyceryl tristearate (GTS), which are administered as a suspension in diluent. Moxidectin is a chemically modified fermentation product of *Streptomyces yanogriseus noncyanogenus*. GTS is a naturally occurring triglyceride component of eukaryotic cell membranes that, when purified, forms a waxy solid at physiologic temperature. Microspheres containing 10% moxidectin are prepared by dissolution of moxidectin in melted GTS to a concentration of 10%, and the solution is processed to the finished microspheres using standard manufacturing technologies. These are packaged, sterilized by gamma-irradiation, and marketed with a specific volume of diluent appropriate to support the intended dose rate for PH 6 (0.17 mg/kg) or PH 12 (0.5 mg/kg). The ingredients (moxidectin, GTS) and manufacturing process are carefully defined and controlled (compendial grade materials; good manufacturing practice controls) to assure a uniformly consistent product composition and quality. Chemical assay of irradiated microspheres showed that sterilization by gamma-irradiation did not produce meaningful quantities of impurities [2].

Following subcutaneous injection, the diluent quickly diffuses away, leaving the microspheres behind in the tissue space at the injection site. Local tissue hydrolytic processes begin acting slowly on GTS, yielding 2-monoacylglycerol, stearate and glycerol, all of which are soluble in aqueous conditions; and diacylglycerol, which is less soluble [3]. All are identical to the naturally occurring hydrolysis products of endogenous GTS, which are eventually metabolized by beta-oxidation in mitochondria or re-used in cellular biosynthesis. As GTS is eroded, moxidectin is liberated from the microspheres, redistributed *via* plasma to tissue repositories elsewhere, and is eventually eliminated unchanged in feces [4].

The safety margin of moxidectin in normal dogs had already been established in terms of systemic moxidectin exposure. When administered to Beagle dogs for one year at a daily dietary intake of 45 ppm in feed (estimated dose 1.13 mg/kg/day), moxidectin was well tolerated [5]. In a follow-on study, after the same oral dosage was administered for one month, estimated maximal concentration (C_max_) was 490 ng/ml [6]. In contrast, when moxidectin was administered to laboratory Beagles as PH microspheres at 0.5 mg/kg subcutaneously, C_max_ was 50-fold lower at 9.37 ng/ml [7]. Other safety objectives, including safety in reproducing male and female dogs, heartworm-positive dogs, and ivermectin-sensitive Collies, had been established prior to suspension of the original development programme. The remaining safety objectives derived from identification of the origin and incidence of hypersensitivity-related responses. A literature search revealed that direct attempts at both prospective and retrospective laboratory investigation of these responses have many of the same limitations as was the case 35 years ago [8–11] and most remain idiosyncratic. In the absence of options for direct investigation, some indirect evaluations were attempted. At the site of injection, the tissue response and the morphologic characteristics of degrading microspheres were carefully evaluated for the type and character of inflammation. Due to the anticipated low incidence of these reactions, the study of McTier et al. [1] was doubled in size with hope that hypersensitivity-related reactions might be observed and more carefully described.

This article describes five laboratory studies conducted to assess the safety of PH 12. Safety margin in chronic use (Study 1) was evaluated by a series of clinical, laboratory and pathology evaluations at multiple dose levels. Safety in reproducing male and female dogs (Studies 2 and 3, respectively) was evaluated by administering PH 12 at times coinciding with key reproductive and developmental phases and monitoring reproductive indices. Safety in heartworm-positive dogs (Study 4) was evaluated by monitoring clinical and laboratory signs, followed by confirmation of adult worm burdens by necropsy evaluation. Due to the chemical similarity of moxidectin to ivermectin, the safety of PH 12 was also evaluated in ivermectin-sensitive Collies (Study 5). Supplementary safety evaluations derived from the field safety and effectiveness study [1] and from a series of expert reports, each prepared to address specific outstanding questions from USA regulatory authorities about PH 12.

## Methods

Five laboratory studies were conducted to demonstrate the safety of PH 12 in dogs. All specialized procedures within the studies were overseen by subject matter experts who produced study reports reflecting their respective subject matter expertise. The expert reports will be briefly summarized in the “Discussion” section as they extend the interpretation of target animal safety of PH 12 in dogs.

For all studies, PH 12 was obtained as a commercial product directly from the Zoetis manufacturer. Studies 1 to 4 used purpose-bred laboratory Beagle dogs sourced from Covance Research Products (Cumberland, VA, USA; Studies 1–3) or from Liberty Research (Waverly, NY, USA; Study 4). Study 5 used ivermectin-sensitive Collies obtained from two commercial suppliers (kennels of Mrs Virginia Lane, Allegan, MI and Ms Janet Nickerson, Leeds, ME). All dogs were healthy on physical examination and current on vaccinations when studies were initiated. Assignment of animals to treatments, cages, and rooms (where applicable) was randomized. Each dog was housed according to guidelines for cages, lighting, humidity and temperature [12]. Animal observations occurred at least once or twice daily, and observations and daily maintenance procedures included social interactions with the animal attendants. Food and water were offered *ad libitum*.

### Study design

#### Study 1: margin of safety

A total of 32 dogs (16 male; 16 female), 6–7 months of age, were divided equally among four treatment groups consisting of either control (0.9% sodium chloride (saline) solution for all studies) or PH 12 at 0.5 mg/kg (1×), 1.5 mg/kg (3×) or 2.5 mg/kg (5×). Treatments were administered three times at 6-month intervals (half the recommended 12-month dose interval), and the study was terminated on Day 379.

#### Study 2: male reproductive safety

Mature male dogs (*n* = 16), 29–37 months-old and with normal pretreatment semen characteristics, were divided equally into two treatment groups, control or 1.5 mg/kg (3×) PH 2. Treatment was administered on Day 1 and the study was terminated on Day 92.

#### Study 3: female reproductive safety

Forty female dogs, known to be brucellosis-free and proven fertile by having birthed at least two litters that exhibited low pup mortality and no abnormalities that would result in death or warrant euthanasia, were divided equally into five groups. One group of dogs served as the control (untreated) group, and the other four groups received a single dose of PH 12 (1.5 mg/kg (3×)) at one of the following time periods corresponding to key reproductive and/or developmental time points: approximately 1 month prior to anticipated mating (premating), 1 day after first tie (gestation Day 2), 28 days after the first tie (gestation Day 29), or 5 days after completion of parturition (lactation Day 6). The 20 breeding males used in this study were left untreated.

Mating was managed as follows. Females were monitored for the onset of heat and placed in the male’s cage 7 days after heat was detected. Following observation of a successful mating (tie; designated Day 1 of gestation) the female was returned to her own cage. Two days later (Day 3 of gestation), the procedure was repeated, and the animals were observed for successful mating. If mating was not observed, the females were returned to the male’s cage daily until a tie was observed or until vaginal cytology indicated she was out of estrus. If the pair were not compatible or did not mate after 3 days, an alternate male was selected.

#### Study 4: safety in heartworm-positive dogs

Sixteen dogs (8 male; 8 female), 7 to 17 months of age and surgically implanted with adult heartworms, were randomly divided into 2 groups per sex (*n* = 4). To confirm study eligibility prior to treatment allocation, heartworm infection was verified with a positive female adult heartworm antigen test (DiroChek^®^ Heartworm Antigen Test Kit, Zoetis) and by the presence of circulating microfilariae. On Day 1, dogs received either saline (control) or 1.5 mg/kg (3×) PH 12 subcutaneously.

#### Study 5: safety in ivermectin-sensitive dogs

Collies (6 male, 9 female) ranging in age 7 to 78 months, previously demonstrated sensitive to a dose of 120 μg/kg ivermectin, were randomly divided into 3 groups (*n* = 5). Each group was treated once with PH 12 at 0.5, 1.5 or 2.5 mg/kg.

### Observations

All persons who made any subjective observations during the in-life phases of studies were unaware of the treatment allocation until completion of data collection. In each study, dogs were observed for abnormalities including, but not limited to, behavioral, neurological, respiratory, or gastrointestinal signs at least once (Study 2) or twice (all other studies) daily. Only Studies 1 (Margin of safety) and 4 (Safety in heartworm-positive dogs) required euthanasia of dogs and post-mortem tissue collection. For each of the gross necropsy phases of these studies, the pathologists or persons performing necropsies did not know the treatment allocation until after completion of that phase.

#### Study 1

For this study, the end points closely followed VICH GL43 [13], with details further specified as follows. All dogs were observed at least twice daily for general health, beginning with arrival at the facility and continuing throughout the in-life phase of the study. On each day of dosing, animals were observed continuously beginning prior to dosing and for approximately 30 min post-dosing. Clinical observations by a veterinarian occurred prior to dosing, 30 min post-dosing, and 1, 2, 4, 8, 24 and 48 h post-dosing. Physical examinations by a veterinarian were conducted prior to dosing, and on Days 92, 183 (pre-dose), 274, 365 (pre-dose) and 379. Injection sites were permanently marked so they could be relocated, with each being examined visually and by manual manipulation on Days 1 (pre-dose), 8, 57, 120, 183 (pre-dose), 190, 239, 302, 365 (pre-dose) and 372. If present, injection site reactions were graded using a modified Draize technique [14]. Post-mortem, injection sites were measured for length, width and depth. Body weight was measured weekly for the first 14 weeks of the treatment phase, then every 4 weeks thereafter; additional body weights were measured 3 times prior to entering the treatment phase, as well as prior to each treatment, and at the end of the in-life phase. Feed consumption was measured daily beginning 1 week prior to the first dosing and continuing throughout the treatment phase. Any instance of an abnormal health observation made at any other time was referred to the study veterinarian for a follow-up examination.

Serial clinical pathology evaluations consisted of hematology, serum chemistry, coagulation profile (prothrombin time, partial thromboplastin time), and urinalysis (physical characteristics, dipstick, sediment examination). For these assays, blood and urine specimens (voided overnight) were obtained from animals after an overnight fast, twice prior to each dosing, and on Days 57, 120, 181, 239, 302, 363 and 378 during the treatment period. Blood samples for pharmacokinetic analysis were collected on Days 1, 2, 4, 11, 31, 46, 60, 91, 121, 183, 184, 186, 193, 213, 228, 242, 272, 303, 365, 366, 368 and 375 and assayed using liquid chromatography with tandem-mass spectrometry (see Additional file 1: Text S1).

On Day 379, following humane euthanasia and exsanguination, complete necropsies were conducted under the supervision of a board-certified veterinary pathologist. A comprehensive set of tissues was collected in accordance with VICH GL43 [13] and placed into 10% neutral buffered formalin or other appropriate fixative (see Additional file 1: Text S1). Fixed tissues were processed to tissue blocks and hematoxylin and eosin-stained slides, and then slides were evaluated microscopically by a board-certified veterinary pathologist. Dose sites from the Day 1 injection site were collected from all dogs in the 0.5 mg/kg moxidectin group and assayed by liquid chromatography tandem mass spectrometry for residual moxidectin content (see Additional file 1: Text S1). Statistical methods are presented in Additional file 1: Text S1.

#### Study 2

Physical examinations were performed prior to dosing, on Day 36, and at the end of the in-life phase (Day 92). Injection site reactions were monitored visually and manually at time of physical examination.

Semen quality of each dog was determined on ejaculates of semen taken pretreatment, and on Days 8, 22, 36, 43, 64, 78, and 92 post-treatment. Semen quality was characterized by scoring for sperm motility, morphology, semen volume and semen concentration. Total sperm count and the number and type of any abnormality were recorded. Statistical methods are presented in Additional file 1: Text S1.

#### Study 3

All female dogs were given a physical examination prior to study initiation and weekly thereafter, with the final exam taking place following completion of parturition (lactation Day 1). The number and date(s) of mating of each treated dog, qualitative gestational events, parturition, litter size, and congenital abnormalities were recorded. Progeny produced during Study 3 were given physical examinations and weighed on lactation Days 2, 5, 8, 15, 22, 29, 36 and 43. A gross necropsy and tissue collection for further evaluation were only completed for dogs or puppies that died spontaneously or were euthanized *in extremis*. Safety was evaluated based on the above reproductive indices and on progeny health. Statistical methods are presented in Additional file 1: Text S1.

#### Study 4

Physical examinations were conducted prior to dosing and on Days 3, 15, 29 and 57. Clinical observations were made at the following times after the last animal was treated: 15 min, then 2, 4, 8, 12 and 24 h. Injection site reactions were monitored visually and manually as part of the physical examination.

Anticoagulated blood was collected for heartworm antigen (DiroChek^®^ Heartworm Antigen Test Kit) and microfilariae evaluation prior to treatment allocation for study eligibility confirmation and again on Days 2, 4, 8, 15, 22, 29 and 43 post-treatment. Microfilaria evaluations were performed by Giemsa staining of a microscope slide bearing a smear of dried lysate from 20 μl whole blood, followed by counting under a light microscope. If no microfilariae were found, then 1-μl anticoagulated whole blood from the same sample was processed *via* modified Knott’s test for counting of microfilariae. On Day 43, following humane euthanasia, the pleural cavity was examined grossly for adult heartworms, with further dissection and examination of tissues occurring in the following order: heart, lungs, and pulmonary arteries. Individual heartworms were sexed, enumerated, measured for length, evaluated for viability based on normal appearance and motility, and then placed in 10% neutral buffered formalin.

#### Study 5

Dogs were monitored for clinical signs of avermectin toxicosis as follows. Physical examinations and clinical observations were made the day before treatment, and additional clinical observations were made 1, 2, 3, 4, 5, 6, 7, 8, 12, 18, 24 and 30 h post-treatment. Dogs continued to be monitored daily for 7 days post-treatment.

### Data analyses and reporting

All specialized procedures within the studies were overseen by subject matter experts, who then produced study reports summarizing and interpreting their data and reflecting their subject matter expertise. All statistical analyses of treatment effect were considered significant at or below the 10% level for Studies 1 and 2 unless otherwise noted. Study 3 reported all results at the 5% and 1% levels (after Bonferroni correction), and Study 4 considered treatment effect statistically significant at the 5% level. Data were transformed where necessary to better meet the assumptions of the statistical procedures.

#### Study 1

Clinical observational comments were summarized. Descriptive statistics, such as arithmetic mean, median, standard deviation, minimum value, and maximum value, were summarized for all continuous variables. All clinical observations were summarized by time point using frequency distributions. Body weight, monthly average daily feed consumption, and quantitative clinical pathology data were analyzed by a general linear mixed model for repeated measures. Organ weights and organ weights relative to final body weight or brain were analyzed using a mixed linear model. For statistically significant effects, pairwise comparisons were performed without regard for controlling error inflation, and least squares means with 95% confidence intervals were calculated. Pathology data were compiled, tabulated, and reported along with a board-certified veterinary pathologist’s separate interpretive summary of findings. The pathologist’s report underwent a formal pathology peer review [15]. Clinical pathology results and statistical outcomes were evaluated by a board-certified veterinary clinical pathologist, who issued an interpretive report of the results.

A trained pharmacokineticist used plasma moxidectin concentrations to calculate pharmacokinetic parameters. These were statistically analyzed using non-compartmental analysis. Estimates of area under the plasma concentration versus time profile were determined using trapezoidal summation. The following variables were calculated for each dose to each dog: area under the plasma concentration versus time curve from time 0 to time t (AUC_0-t_), C_max_, and time to maximal concentration (t_max_). A mixed linear model for repeated measures was used to determine means and confidence intervals for the pharmacokinetic variables.

#### Study 2

Simple regression analysis was used on all parameters by treatment group, and the coefficients of regression were compared by Z-test. A mixed model with repeated measures analysis was also performed. Least-squares means for each parameter were compared between treatment groups using a Student’s t-test.

#### Study 3

Group pairwise comparisons were used to compare female body weights, female body weight changes, gestation length, litter size, number of live pups, and pup stillborn index. A fertility index, mating index, pregnancy index, and gestation index were compared to control by a two-tailed Fisher’s exact test with a Bonferroni correction. Pup sex ratio was analyzed using a Chi-square test for homogeneity with sex as the row variable and treatment group as the column variable. Mean pup weight was summarized by descriptive statistics and analyzed by covariate analysis with litter size as the covariate when conducting Dunnett’s test.

#### Study 4

Microfilaria counts from each dog for each observation were analyzed by 2-way analysis of variance (ANOVA). Similarly, at necropsy, live and dead heartworms by sex of heartworm were analyzed. For each day, least squares means for the various parameters in the 2 treatment groups were compared by a one-way ANOVA F-test.

#### Study 5

This study consisted solely of clinical observations and did not require any statistical analysis.

## Results

### Study 1

The only treatment-related finding was at injection sites, where very slight or slight swelling was observed in some, but not all, dogs in each treatment group (Table 1). In the 0.5 mg/kg group, injection site swelling was identified only after the Day 183 injection, in some but not all dogs; and swellings gradually became non-detectable. In the groups in which dogs received the highest dose volumes (1.5 mg/kg and 2.5 mg/kg groups), swellings were identifiable more consistently following treatment, and some of these swellings could be re-located 6 to 12 months later.

**Table 1:** Clinically detectable injection site swelling

Injection site	Group^a^	Degree of swelling^b^	Day of study										
			1	8	57	120	183	190	239	302	365	372	379
Day 1	Control^c^	None	6	6	8	8	8	8	8	8	6	8	8
		Very slight	2	2	–	–	–	–	–	–	2	–	–
	0.5 mg/kg	None	–	8	8	8	8	8	8	8	8	8	8
	1.5 mg/kg	None	–	8	8	6	5	4	6	6	1	1	4
		Very slight	–	–	–	2	2	2	1	–	3	3	3
		Slight	–	–	–	–	1	2	1	2	4	4	1
	2.5 mg/kg	None	–	6	7	7	3	7	7	4	1	1	5
		Very slight	–	2	1	1	3	–	–	2	2	3	1
		Slight	–	–	–	–	2	1	1	2	5	4	2
Day 183	Control	None	–	–	–	–	8	7	7	8	7	8	8
		Very slight	–	–	–	–	–	–	–	–	1	–	–
		Slight	–	–	–	–	–	1	1	–	–	–	–
	0.5 mg/kg	None	–	–	–	–	7	8	8	8	6	5	8
		Very slight	–	–	–	–	–	–	–	–	2	2	–
		Slight	–	–	–	–	1	–	–	–	–	1	–
	1.5 mg/kg	None	–	–	–	–	8	6	6	5	3	2	4
		Very slight	–	–	–	–	–	1	1	–	1	2	4
		Slight	–	–	–	–	–	1	1	3	4	4	–
	2.5 mg/kg	None	–	–	–	–	8	8	8	3	1	1	3
		Very slight	–	–	–	–	–	–	–	4	2	1	3
		Slight	–	–	–	–	–	–	–	1	5	6	2
Day 364	Control	None	–	–	–	–	–	–	–	–	–	–	8
	0.5 mg/kg	None	–	–	–	–	–	–	–	–	–	–	8
	1.5 mg/kg	None	–	–	–	–	–	–	–	–	–	–	3
		Very slight	–	–	–	–	–	–	–	–	–	–	4
		Slight	–	–	–	–	–	–	–	–	–	–	1
	2.5 mg/kg	None	–	–	–	–	–	–	–	–	–	–	3
		Very slight	–	–	–	–	–	–	–	–	–	–	3
		Slight	–	–	–	–	–	–	–	–	–	–	2

^a^Control animals 0.9% sodium chloride (saline) solution. All other animals received ProHeart® 12. *n* = 8 for all groups

^b^‘Very slight’ is defined as ‘barely perceptible’. ‘Slight’ is defined as ‘edges of area well-defined by definite raising’

–, no dogs with this categorization

Macroscopic observation of moxidectin injection sites at necropsy revealed focal subcutaneous thickening, considered related to moxidectin treatment, ranging in size up to 15 mm long, 12 mm wide and/or 3 mm thick for the 0.5 mg/kg moxidectin group, and 40 mm long, 25 mm wide and/or 8 mm thick in the 1.5 and 2.5 mg/kg moxidectin groups. Microscopically, thickened areas were observed to have mild to marked granulomatous inflammation and variable aggregates of rounded spaces (microspheres).

The percent residual moxidectin, contained in the injection site of select animals from the 0.5 mg/kg, ranged from 0.045 to 37.6% of dose (1.8–1506 μg) with an average of 16.0% residual moxidectin (Table 2). Other than injection sites, there were no clinically meaningful differences between treated groups and controls with respect to clinical signs, body weight, food consumption, or any of the clinical pathology tests. All findings were considered incidental and characteristic of dogs of that age.

**Table 2:** Residual moxidectin in injection sites one year after 0.5 mg/kg dose

Animal number	Sex	Dose administered (μg)	Residual moxidectin (μg)	Dose remaining in injection site (%)
1	M	4000	4.4	0.109
2	M	5000	1227	24.5
3	M	5000	1445	28.9
4	M	5000	34.0	0.679
5	F	4000	1.8	0.045
6	F	4000	586	14.6
7	F	4000	842	21.0
8	F	4000	1506	37.6

*Abbreviations*: F, female; M, male

**Fig. 1:**
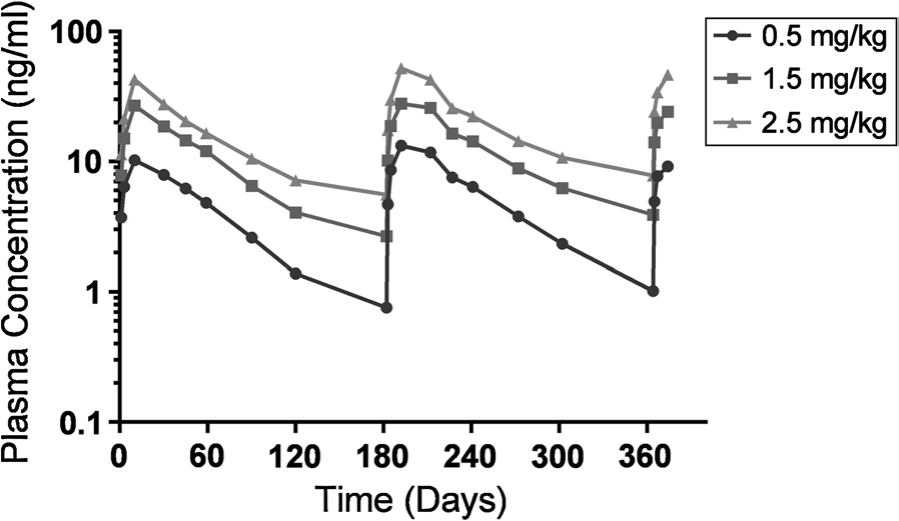
Moxidectin mean plasma concentrations in dogs administered 1×, 3×, and 5× the recommended 0.5 mg/kg dose at 6-month intervals

Pharmacokinetic analysis demonstrated that AUC_0-t_ and C_max_ increased in a dose-proportional manner from 0.5 mg/kg to 2.5 mg/kg following the first dose and continued for subsequent doses. The observed C_max_ values for subsequent doses appear to show little or no accumulation (Fig. 1).

### Study 2

There were no clinically meaningful differences between groups in the various indicators of semen quality (Table 3). There was no significant difference between the moxidectin-treated and control groups when the total volume of ejaculate was analyzed. When only the sperm-rich fraction was considered, statistical analysis indicated that the moxidectin group had a larger sperm rich faction than the control group; however, this finding may have been due to the study procedures for physical separation of the fractions. The percent abnormal midpiece and the percent proximal droplet were significantly lower in the moxidectin group compared to controls over the course of the study. A significant decrease in motile sperm count and percent motility in the moxidectin group on Day 22 was attributed to a difficult collection in 1 treated animal that likely impacted these parameters. Injection site reactions were observed in 4 control dogs from Day 2 to Day 17 and in 6 of the treated dogs from Day 2 to Day 11.

**Table 3:** Relevant sperm parameters

Parameter	Control	0.51 mg/kg moxidectin
Presperm fraction volume (ml)	2.55 ± 0.28	1.63 ± 0.29
Sperm-rich fraction volume (ml)	3.02 ± 0.27	3.36 ± 0.29
Volume per ejaculate (ml)	5.44 ± 1.19	4.99 ± 1.21
pH of ejaculate	6.86 ± 0.06	6.75 ± 0.06
Concentration of sperm (×10^6^)	184.26 ± 30.63	151.07 ± 31.40
Total sperm count (×10^6^)	508.26 ± 0.06	437.23 ± 0.07
Total normal sperm count (×10^6^)	441.22 ± 0.07	391.57 ± 0.07
Percent normal sperm count	83.35 ± 0.89	88.09 ± 0.92
Motile sperm count (×10^6^)	412.20 ± 0.08	343.70 ± 0.08
Percent motility	76.11 ± 2.07	75.18 ± 2.15
Percent progressive motility	82.41 ± 3.13	79.91 ± 3.28
Percent abnormal head	1.80 ± 0.26	1.84 ± 0.27
Percent loose head	1.93 ± 0.65	1.96 ± 0.66
Percent abnormal midpiece	7.04 ± 0.88	3.13 ± 0.92
Percent proximal droplet	1.41 ± 0.44	0.86 ± 0.46
Percent distal droplet	4.48 ± 0.86	4.13 ± 0.88

*Note*: All parameters are shown as the arithmetic mean ± SD. *n* = 8 for each group

*Abbreviation*: SD, standard deviation

### Study 3

There were no treatment-related clinical signs or differences in body weight when comparing the treated with control mothers. From whelping through lactation, mothers showed a normal initial weight loss followed by maintenance or weight gain. All females (8 of 8) treated with moxidectin at Gestation Day 29 and 7 of 8 in controls and other moxidectin groups conceived and whelped. One dog (moxidectin in premating period) had a prolonged labor but responded to oxytocin. Another (moxidectin on Gestation Day 2) had a prolonged gestation but whelped normally.

Pup survival was overall excellent; incidence of stillbirths and neonatal mortality was low overall, distributed without relationship to treatment. Each of the treatment and control groups had totals of 37 to 46 pups born and 1 to 4 deaths. Among pups that did not survive, there were no lesions to suggest a test article-related effect. All litters in treated and control groups completed the 6-week lactation interval, showing no differences between treated groups and controls with respect to body weight at birth or during lactation (Table 4).

**Table 4:** Summary of reproductive performance in females treated with ProHeart^®^ 12 at 1.5 mg/kg (3× overdose)

Timing of treatment	None (Control)	Pre-Mating	Mating	Mid-gestation	Lactation
Pups per Litter (mean ± SD)	4.3 ± 2.12	5.5 ± 1.85	5.0 ± 1.77	6.1 ± 2.34	6.3 ± 1.96
Liveborn/Total (%)	100	100	100	100	100
Postnatal viability^a^ (% ± SD)	92 ± 17.68	97.9 ± 5.89	100 ± 0.00	93.3 ± 8.64	100 ± 0.00
Birth weight (g) (mean ± SD)					
Males	308 ± 31.6	300 ± 47.62	290 ± 50.91	275 ± 35.40	309 ± 55.46
Females	292 ± 26.30	266 ± 72.34	270 ± 27.98	269 ± 22.29	295 ± 39.90
Weaning weight (g) (mean ± SD)					
Males	2288 ± 134.7	2172 ± 242.8	2170 ± 252.9	2060 ± 172.2	2174 ± 503.9
Females	1996 ± 221.7	1912 ± 310.4	2038 ± 394.0	1914 ± 282.3	1865 ± 330.1

*Note*: No statistical differences were identified in any indices of reproductive performance (*P* > 0.05)

^a^Number liveborn that were weaned on Day 42

*Abbreviation*: SD, standard deviation

### Study 4

A single dose of moxidectin was well tolerated. There were no treatment-related abnormal clinical signs after subcutaneous administration and all dogs survived until the end of the study. One moxidectin-treated dog was microfilaria-negative following induced infections and prior to treatment but was heartworm-positive at the end of study. Significant reductions were observed in the microfilaria count of the moxidectin-treated group compared with untreated controls on Days 4, 8, 15, 22, 29 and 43 post-dose, with microfilarial count reductions of 84.22%, 98.81%, 99.91%, 99.95%, 99.97% and 99.92%, respectively (Table 5). At necropsy, all dogs were found to be heartworm-positive, with significantly lower adult worm counts in moxidectin-treated animals observed compared to control (16.6 *vs* 18.9, respectively; *P* < 0.05, Table 6).

**Table 5:** Geometric mean microfilaria counts (number of microfilariae per ml)

Study day	Control (*n* = 8)	1.50 mg/kg moxidectin (*n* = 7)	% Reduction^a^
Day -70	0	0	
Day -49	6.4	4.8	
Day -35	55.5	113.8	
Day -21	730.6	1743.4	
Day -7	3401.1	4855.3	
Day -2	2648.3	2446.4	
Day 1	4228.8	5622.0	0
Day 4	4393.4	693.1	84.22
Day 8	4828.5	57.4	98.81
Day 15	6271.6	5.6	99.91
Day 22	9269.0	4.6	99.95
Day 29	7413.1	2.5	99.97
Day 43	5186.0	4.2	99.92

^a^Percent reduction is compared to controls at the same time point

**Table 6:** Geometric mean worm counts

Worm count	Control	1.50 mg/kg moxidectin
*n*	8	8
Males	9.5	8.2
Females	9.4	8.3
Total	18.9	16.6^a^

*Note*: Each dog had been given an adult heartworm infection (10 adult male and 10 adult female heartworms) by surgical implantation 70 days prior to treatment with control or test article

^a^*P* < 0.05 compared to control

### Study 5

There were no adverse reactions in ivermectin-sensitive Collies resulting from administration of PH 12 doses of 0.5 mg/kg, 1.5 mg/kg, or 2.5 mg/kg moxidectin.

## Discussion

The five laboratory studies and the field safety study supporting the safety evaluation of PH 12 constitute a typical investigational programme for a long-acting veterinary pharmaceutical product administered subcutaneously. Study 1 was an adaptation of the traditional margin of safety study [13]. Doses used were 1, 3 and 5 times the recommended 0.5 mg/kg dose. The nominal dose interval is 1 year, which is considered essentially a “single-dose product.” The recommended 1-year interval (0-, 1-, and 2-year time points) would make the study much longer than the 6–9 months duration that typically provides all the information essential to safety evaluation of a chronically administered product [16, 17] already known to be tolerated at C_max_ > 50-fold higher than anticipated for PH 12 [5]. Ultimately, dosing days and study duration were chosen to address objectives related to regulatory questions: pharmacokinetics of moxidectin over an extended time, quantifying the amount of moxidectin remaining at the 1× injection site after one year, and serial evaluations of injection sites for evidence of a local hypersensitivity-related response.

Study 1 outcomes were consistent with the very wide calculated safety margin for moxidectin in PH 12; there were no suspected pharmacologic effects of moxidectin. Study 1 pharmacokinetics extend the basis for inference by showing both dose proportionality and no systemic accumulation even at a 5× overdose and the 6-month rather than the 12-month dose interval. Residual moxidectin at 1× injection sites ranged from near zero to 37.6%. At injection sites, the only clinically evident treatment-related findings were persistent, non-adverse small swellings, as expected. Swellings from the 3× and 5× groups (dose volumes ≥ 1.1 ml) could still be found after one year. Microscopically, the swellings consisted of microspheres surrounded by granulomatous inflammation, without evidence of a local hypersensitivity-related response. The microscopic appearance did not differ markedly over time or with the range of doses administered. There were no local or systemic hypersensitivity-related responses of any kind in the study. The wide margin of safety was also demonstrated in ivermectin-sensitive Collies in Study 5.

Studies 2 and 3 demonstrated the safety of moxidectin in reproducing male and female dogs. In Study 4, heartworm-positive dogs showed good toleration of a single dose of moxidectin. Neither the marked post-treatment drop in microfilaria counts nor the lower numbers of adult worms (including 1 dog with identifiable worm fragments) were associated with any clinical signs. However, in other studies, clinical signs have been seen associated with die-offs of microfilariae or adults. In one study [18], PH 12 administered at four months but not six months after inoculation with infective larvae was shown to reduce counts of adult heartworms > 80%. In the week following the first dosing, dogs dosed at four months post-inoculation, (but not dogs dosed at 6 months post-inoculation) showed an increase in the incidence of emesis and abnormal feces (e.g. single instances of soft mucoid feces, sometimes dark-colored or blood-tinged). There were no other such clusters of observations during the study. One consequence of adulticidal or microfilaricidal activity is an acute-phase protein response [19], associated with systemic release of heartworm-associated antigens. Current guidelines for management of heartworm-positive dogs [20] incorporate doxycycline therapy and limited but repeated adulticidal therapy to reduce the absolute numbers of parasites or microfilariae killed at any one treatment event. The associated reduction in release of total parasite-related antigen (*D. immitis*, *Wolbachia* spp.) [21] contributes to a reduction in the incidence and severity of shock-like adverse clinical signs associated with treatment [22].

Further to the above laboratory studies, histology sections of injection sites from earlier PH studies were re-evaluated [23] by a board-certified veterinary pathologist for suggestive evidence of hypersensitivity-related responses and to evaluate microsphere spaces for possible evidence of degradation by any unexpected means. Digital image analysis of microspheres (voids in pathology sections) at multiple time points post-injection was used to evaluate residual microspheres and support evaluation of microsphere degradation. The microscopic appearance of injection sites was the same granulomatous inflammation as described in Study 1. Changes approximately one month post-injection were considered features of a tissue healing reaction to a non- or minimally-immunogenic material, while later changes were considered features of the continuing presence of the material but not of active inflammation. The progression is consistent with a reparative tissue response (wound healing response) in the presence of a non- or minimally-immunogenic biodegradable material that is too big to be phagocytosed by macrophages. The tissue response is consistent with extensive literature of the safety evaluation of implanted biomedical devices or tissue fillers [24], where the observed changes have been well characterized as resolving rather than pro-inflammatory or hypersensitivity-related. Image analysis of microsphere voids showed changes consistent with surface-erosion down to ~ 30 μm. The absence of smaller diameters was presumed due to phagocytosis and final destruction of microspheres. The results of image analysis are consistent with a lipolysis model of similar microspheres, where lysozyme was steadily released from the dissolving surface of GTS microspheres by diffusion [25].

A further effort to understand hypersensitivity-related reactions was by increasing the size of the McTier et al. veterinary patient study [1]. By increasing the likelihood of observing a hypersensitivity-related response, the intent was to obtain a comprehensive description and a better understanding of the events. The increased size was decided as follows. From pharmacovigilance reporting of PH 6, a closely related product, between 2008–2016, the estimated incidence of hypersensitivity-related reactions was approximately two dogs per 10,000 doses distributed [26], which is in line with estimates for many marketed human pharmaceuticals [27]. Mathematical simulations based on that estimate showed that in a prospective study, group sizes capable of providing statistically significant estimates of incidence (e.g. 2–8000 per group) were not feasible. However, if the actual incidence were 10- to 40-fold higher than the pharmacovigilance-based estimate, then a study of 200 animals per group would have a 30–80% likelihood of producing ≥ 1 hypersensitivity-related event (J. Boucher, personal communication to M Krautmann). On that basis, Zoetis decided to more-than-double the number of cases enrolled in the field safety and effectiveness study. In fact, 296 dogs were treated monthly with an oral ivermectin:pyrantel combination (control product) and 297 dogs were treated yearly with PH 12 (all 297 dogs received 1 dose and 269 of the 297 dogs received 2 doses). Overall, adverse events occurred without meaningful differences between treatment groups. Hypersensitivity-related reactions considered likely related to treatment included one case for the control product and two cases for PH 12; all three were considered mild to moderate in severity. For the control product, one dog developed erythema and urticaria about the abdomen, axilla, and pinna following oral administration of the first dose. Initial owner treatment with an oral antihistamine was not effective. The dog was re-examined the next day by the veterinarian due to onset of listlessness and vomiting. The veterinarian prescribed a tapering dose of corticosteroids. At follow-up visits, erythema and urticaria were still observed 5 days post-treatment but resolved by 14 days post-treatment. Due to the reaction, the dog was discontinued from the study. For PH 12, the two cases were as follows: several hours after receiving its first treatment, one dog developed hives and swelling about the face and neck. The owner administered oral antihistamine, and the signs were resolved by the following day. At the second dosing a year later, the dog’s treatment was uneventful. The second dog developed swelling about the face and paws, also several hours after dosing. The dog subsequently showed vomiting, polydipsia, and tachycardia. The dog was re-examined at the veterinary clinic and administered an antihistamine. Symptomatic oral follow-up medications were dispensed for home administration. All signs resolved by Day 4. At its second dosing a year later, the dog was administered an oral antihistamine prior to treatment, and the dog’s post-treatment response was uneventful. Five other PH 12-treated dogs and 3 other dogs on the control product were reported with hypersensitivity-related reactions that did not occur in close proximity to treatment during the study. For the PH 12 cases, clinical signs were linked to either thyrotoxicosis, pre-existing dermatological disorders and their treatment, or suspected envenomation. For the control product cases, clinical signs were linked to immunotherapy, vaccination, or dermatological disorders.

The two mild cases of hypersensitivity-related reactions in 297 moxidectin-treated patients is higher than the pharmacovigilance-based report rate, but this is unsurprising. It is well understood that with an unfamiliar drug, practitioners are more likely to report the first one or two adverse reactions. Once they become familiar with the drug, they tend not to report subsequent reactions [28]. From a clinical perspective, the proper context for interpretation of the 2 PH 12 cases is the positive control group, an oral ivermectin/pyrantel combination product, where a similar incidence and severity of such reactions (1 mild case) was observed.

The two PH 12-treated dogs with hypersensitivity-related reactions linked to treatment both had prior exposure to avermectins, suggesting the possibility of an actual acquired immune hypersensitivity response (Type 1). However, the lack of a response after the second PH 12 injection is inconsistent with Type 1 hypersensitivity, suggesting instead that the initial response was anaphylactoid. Such reactions are like Type 1 hypersensitivity reactions in that the same inflammatory cells are activated, the same mediators are released from granulocytes, onset is within minutes of dosing, and responses can be mild to fatal. The difference is that in anaphylactoid responses, inflammatory cells or mediators are activated through direct drug effects and mechanisms not involving antibody-receptor interaction. Therefore, anaphylactoid reactions can be observed following administration of the first dose of the agent [29], and they may not occur at subsequent exposures. In a recent international consensus document on drug allergy, the authors stated that “when drug reactions resembling allergy occur, they are called drug hypersensitivity reactions (DHRs) before showing the evidence of either drug-specific antibodies or T cells” [8]. Furthermore, this premature and often incorrect diagnosis can cause clinicians to unnecessarily withhold treatment [9, 30]. In the position paper, the authors concluded that the diagnosis of DHRs is often challenging and requires the same careful approach, no matter which specific drug is involved. Diagnosis remains largely clinical, with the help of certain allergy tests that are available for some of the drug classes; and new and validated biological tests for diagnosis, available to all clinicians, are necessary to improve care for these patients [8].

Regarding the origin of hypersensitivity related responses, none of the findings in literature or any of the *in vivo*, *in vitro* or chemical approaches were helpful. A pharmacovigilance-based estimated incidence of around two hypersensitivity-related cases (combined anaphylaxis and anaphylactoid, the latter much more common) per 10,000 doses distributed is in line with other marketed products in human medicine [27]. In the study of McTier et al. [1] where PH 12 was compared with an orally administered ivermectin-pyrantel control, there were three mild to moderate anaphylactoid responses observed; one in the positive control product and two in the PH 12 group. All three had similar clinical presentations and responses to treatment. Neither PH 12-treated dog had any reaction a year later when PH 12 was re-administered. Thus, both the pharmacovigilance-based estimate and the veterinary patient study outcome suggest that the incidence of hypersensitivity-related responses for PH 12 is in line with that of other marketed products. It is difficult to imagine otherwise for a product that has been so well accepted in non-USA markets for so long [31].

## Conclusions

The safety of PH 12 (GTS microspheres containing 10% moxidectin) was evaluated in a series of laboratory studies and a large veterinary patient study. The laboratory studies demonstrated a wide safety margin, safety in reproducing adult male and female dogs, safety in heartworm-positive dogs, and safety in ivermectin-sensitive Collies. The studies included demonstration of pharmacokinetics, injection site residues after one year, in-depth evaluation of local tissue toleration, and image analysis-based characterization of the performance of the microspheres. Injection of PH microspheres causes a local tissue response characteristic of a biocompatible medical device and creates a tissue repository from which moxidectin is released steadily and gradually by surface erosion. The rate of release of moxidectin into the systemic pool is slower than the rate of its systemic clearance (uptake-limited pharmacokinetics), thereby preventing systemic accumulation even at much higher dose multiples than the therapeutic dose. Surface erosion of microspheres proceeds by hydrolytic processes from adjacent cells that eventually reduce the size of microspheres until they are phagocytosed at approximately 30 µm. After all microspheres have been phagocytosed, an area of fibrosis remains. Residue data from injection sites after one year reflect differences between dogs in rate of microsphere erosion, but with clear pharmacokinetic evidence that there is no systemic accumulation, the presence of residual microspheres from last year’s dosing has no bearing on yearly decisions to re-dose. The origin and incidence of hypersensitivity-related responses were not directly evaluable. Indirect investigations in a variety of areas including effects of irradiation, local tissue response, and microsphere erosion/clearance reflected good local toleration and nothing unexpected or suggestive of a problem. Pharmacovigilance monitoring and the outcome of a very large veterinary patient study suggested that such reactions are in line with currently marketed products. Taken together, along with pharmacovigilance monitoring of PH products worldwide over 18 years, these extensive investigations demonstrated the well understood and characterized safety of PH 12 in dogs.

## Supplementary information

**Additional file 1: Text S1.** Additional Methods.

## Data Availability

The datasets supporting the conclusions of this article are included within the article. Due to commercial confidentiality of the research, data not included in the manuscript can only be made available to *bona fide* researchers, subject to a non-disclosure agreement.

## Abbreviations

ANOVA: analysis of variance
AUC_0-t_: area under the plasma concentration versus time curve from time 0 to time t
C_max_: maximal concentration
EDTA: ethylenediaminetetraacetic acid
GTS: glyceryl tristearate
LC-MS/MS: liquid chromatography tandem mass spectrometry
PH: ProHeart^®^
PH 6: ProHeart^®^ 6
PH 12: ProHeart^®^ 12
PV: pharmacovigilance
t_max_: time to maximal concentration
