# Supplementary material for: Safety of an extended-release injectable moxidectin suspension formulation (ProHeart® 12) in dogs
Source: Parasit Vectors. 2019 Sep 6;12:433. doi: 10.1186/s13071-019-3690-6 (PMC6728954; doi:10.1186/s13071-019-3690-6)
Supplement: Supplementary file 1 — Additional file 1: Text S1. Additional Methods. [file 13071_2019_3690_MOESM1_ESM.docx]

# Additional file 1. Text S1. Additional Methods

**Regarding PK analysis in Study 1:**

A Waters Acuity UPLC C8 1.7μm, 2.1 x 50 mm column, heated to 50°C, was used (Waters, Milford, MA) in conjunction with a gradient using 0.3% ammonium hydroxide in water and acetonitrile as the 2 mobile phases. Methanol was used as a strong wash and a 0.3% ammonium hydroxide in water:acetonitrile mixture (70:30) as the weak wash. Mass spectrometry was carried out using moxidectin-d3 as an internal standard and an API 5500 LC-MS/MS system (Sciex Applied Biosystems, Foster City, CA).

**Regarding dose site analysis in Study 1:**

A Waters Acuity BEH C8 100 x 2.1 mm column, heated to 55°C, was used (Waters, Milford, MA) in conjunction with a gradient using 0.1% ammonium hydroxide in water and acetonitrile as the 2 mobile phases. An acetonitrile:methanol:water:isopropanol mix (3:3:2:2) was used as a strong wash and an acetonitrile:water mixture (60:40 + 0.1% formic acid) as the weak wash. Mass spectrometry was carried out using moxidectin-d3 as an internal standard and an API 4000 LC-MS/MS system (Sciex Applied Biosystems, Foster City, CA).

**Regarding collection of tissues in Study 1:**

The following tissues were collected: adrenal glands; aorta; brain; large intestine (cecum, colon, rectum); small intestine (duodenum, jejunum, ileum); dose sites; epididymis; esophagus; eyes and optic nerves; femur and sternum with bone marrow; gall bladder; Peyer’s patch; heart; kidney; lesions; liver; lungs and large bronchi; auxillary, mandibular, mesenteric, and superficial cervical lymph nodes; mammary glands, cervix, uterus, ovaries, and vagina, or prostate and testes; biceps muscle; pancreas; pituitary gland; salivary gland; sciatic nerve; skin and subcutis; spinal cord; spleen; stomach, including the cardia, fundus, and pylorus; thymus; thyroid and parathyroid; tongue; trachea; and urinary bladder. Adrenal glands, brain, heart, kidneys, liver pituitary gland, spleen and either ovaries or testes were weighed. Dose sites were measured for length, width, and depth and photographed by masked necropsy personnel.

**Regarding collection of tissues in Study 3:**

In Study 3, the same organs listed for Study 1 were collected except for minor modifications: dose sites, Peyer’s patches, and the auxiliary and superficial cervical lymph nodes, and ribs with bone marrow, and the tracheobronchial and regional lymph nodes were also included.

**Regarding data analysis in Study 1:**

Regarding the general linear mixed model for body weight, feed consumption, and quantitative clin pharm parameters, the model included treatment, time, sex, and interactions between those effects as fixed effects. Random effects included block within sex, room, a between block interaction, treatment within sex and room, and error. Baseline covariates were included as appropriate. If there was a statistically significant 3-way interaction (e.g., treatment by sex by time), appropriate alternative comparisons by sex to control or at each time point were made or the data were summarized by descriptive statistics only. Least-squares means, 90% confidence intervals, standard errors, minimums, and maximums were calculated. Change across time was graphically displayed.

Regarding the organ weights linear model, organ weights and organ weights relative to final body weight or brain were analyzed using a mixed linear model. Fixed effects included sex, treatment, and a sex by treatment interaction. Random effects included room, block within sex and room, and error.

Pharmacokinetic models:

The models included the fixed effects of sex, treatment, study day, and all interactions, with random effects of room, block within sex and room, block by treatment interaction within sex and room, and error. The correlation structure was chosen among repeated measures with the smallest Akaikie information criterion. To test for dose‑proportionality, it was assumed that the logarithms of the PK parameters (AUC_0-t_ or C_max_) were linearly-related to the logarithm of the dose. Thus, C_max_ and AUC_0-t_ was fit to the model: Log(Y) = log(α) + β•Log(dose), where Y equals C_max_ or AUC_0-t_, α was dependent upon other terms in the model, and β equalled 1 if dose-proportionality held. Thus, β was estimated using a model with the fixed effects of sex and log (dose) and random effects of room, block within sex and room, and error. Dose proportionality was concluded if the 95% confidence interval on the estimate of β included 1 and the test for lack of fit was not significant.

**Regarding data analysis in Study 2:**

The mixed model repeated measures analysis included the average pretreatment measurement as a covariate in the model, tested at the 5% level of significance, and contained treatment, block, treatment by block interaction, day, and treatment by day interaction as fixed effects, with block and block by treatment interaction considered random effects.

**Regarding data analysis in Study 3:**

For each endpoint, a Levene’s test assessed homogeneity of group variance. If Levene’s test was not significant, then a Dunnett’s test compared each group to control. If Levene’s test was significant, then comparisons to control were made using Welch’s test with a Bonferroni correction.
